# *N*′-[(1*E*)-1-(5-Chloro-2-hydroxy­phen­yl)propyl­idene]-4-methoxy­benzohydrazide

**DOI:** 10.1107/S1600536809052878

**Published:** 2009-12-16

**Authors:** Ren-Gao Zhao, Jie Lu, Jian-Guo Chang

**Affiliations:** a Department of Material Science and Chemical Engineering, Taishan University, 271021 Taian, Shandong, People’s Republic of China; b Department of Architecture and Mechanical Engineering, Taishan University, 271021 Taian, Shandong, People’s Republic of China

## Abstract

The title compound, C_17_H_17_ClN_2_O_3_, has a *trans* conformation about the C=N double bond and an intra­molecular O—H⋯N occurs. The crystal structure is stabilized by inter­molecular N—H⋯O hydrogen bonds.

## Related literature

For further details of the chemistry of the title compound, see Carcelli *et al.* (1995 ▶); Salem (1998 ▶).
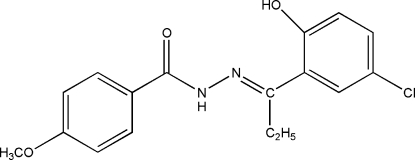


## Experimental

### 

#### Crystal data

C_17_H_17_ClN_2_O_3_
*M* *_r_* = 332.78
Monoclinic, 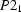

*a* = 8.6313 (13) Å
*b* = 4.9373 (8) Å
*c* = 19.610 (3) Å
β = 102.359 (3)°
*V* = 816.3 (2) Å^3^
*Z* = 2
Mo *K*α radiation
μ = 0.25 mm^−1^
*T* = 295 K
0.25 × 0.17 × 0.12 mm

#### Data collection

Bruker APEXII CCD area-detector diffractometer
Absorption correction: multi-scan (*SADABS*; Sheldrick, 2003 ▶) *T*_min_ = 0.940, *T*_max_ = 0.971
4350 measured reflections
2837 independent reflections
2047 reflections with *I* > 2σ(*I*)
*R*_int_ = 0.024

#### Refinement

*R*[*F* ^2^ > 2σ(*F* ^2^)] = 0.043
*wR*(*F* ^2^) = 0.098
*S* = 1.05
2837 reflections
209 parameters
1 restraint
H-atom parameters constrained
Δρ_max_ = 0.13 e Å^−3^
Δρ_min_ = −0.19 e Å^−3^
Absolute structure: Flack (1983 ▶), 1207 Friedel pairs
Flack parameter: 0.13 (9)

### 

Data collection: *APEX2* (Bruker, 2005 ▶); cell refinement: *SAINT* (Bruker, 2005 ▶); data reduction: *SAINT*; program(s) used to solve structure: *SHELXS97* (Sheldrick, 2008 ▶); program(s) used to refine structure: *SHELXL97* (Sheldrick, 2008 ▶); molecular graphics: *SHELXTL* (Sheldrick, 2008 ▶); software used to prepare material for publication: *SHELXTL*.

## Supplementary Material

Crystal structure: contains datablocks global, I. DOI: 10.1107/S1600536809052878/pk2217sup1.cif

Structure factors: contains datablocks I. DOI: 10.1107/S1600536809052878/pk2217Isup2.hkl

Additional supplementary materials:  crystallographic information; 3D view; checkCIF report

## Figures and Tables

**Table 1:** Hydrogen-bond geometry (Å, °)

*D*—H⋯*A*	*D*—H	H⋯*A*	*D*⋯*A*	*D*—H⋯*A*
O1—H1⋯N2	0.82	1.81	2.526 (3)	145
N1—H1*A*⋯O2^i^	0.86	2.23	2.934 (3)	140

Symmetry code: (i) 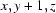.

**Fig. 1.**
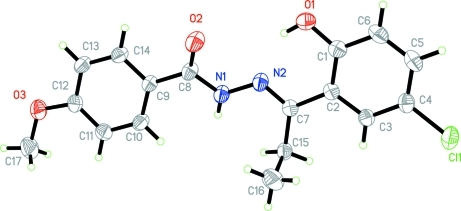
The molecular structure showing the atom-numbering scheme. Displacement ellipsoids are drawn at the 30% probability level.

**Fig. 2.**
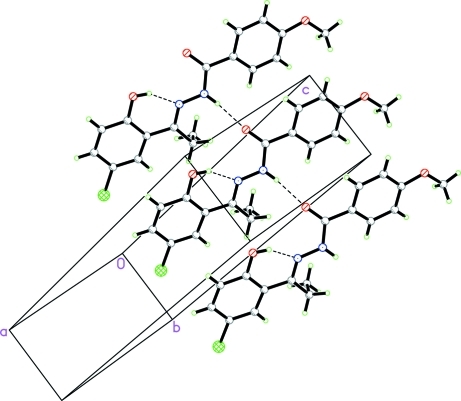
Packing diagram showing intramolecular O—H···N and intermolecular N—H···O hydrogen bonds (dashed lines).

## supplementary crystallographic information

### Comment

The chemistry of aroylhydrazones continues to attract attention due to their coordination ability to metal ions (Salem, 1998) and their biological activity (Carcelli *et al.*, 1995). As an extension of work on the structural characterization of aroylhydrazone derivatives, the title compound was synthesized and its crystal structure is reported here.

The title molecule displays a *trans* conformation with respect to the C7=N2 double bond (Fig. 1). The dihedral angle between the two benzene rings is 3.0 (2)°. The crystal structure is stabilized by intramolecular O—H···N and intermolecular N—H···O hydrogen bonds. (Table 1. and Fig. 2).

### Experimental

4-methoxybenzohydrazide (0.01 mol,1.66 g) was dissolved in anhydrous ethanol (50 ml), and 1-(5-chloro-2-hydroxyphenyl)propan-1-one (0.01 mol, 1.84 g) was added.  The reaction mixture was refluxed for 4 h with stirring, then the resulting precipitate was collected by filtration, washed several times with ethanol and dried *in vacuo* (yield 87%). The compound (1.0 mmol, 0.33 g) was dissolved in dimethylformamide (15 ml) and kept at room temperature for 30 d to obtain colourless single crystals suitable for X-ray diffraction.

### Refinement

All H atoms were positioned geometrically and treated as riding on their parent atoms, with C—H (methyl) = 0.96 Å, C—H (methylene) = 0.97 Å, C—H (aromatic) = 0.93 Å, O—H = 0.82 Å, N—H = 0.86 Å and with *U*_iso_(H) = 1.5*U*_eq_ (C_methyl_, O) and 1.2*U*_eq_ (C_aromatic_, C_methylene_, N).

### Crystal data

**Table:**

C_17_H_17_ClN_2_O_3_	*F*(000) = 348
*M**_r_* = 332.78	*D*_x_ = 1.354 Mg m^−^^3^
Monoclinic, *P*2_1_	Mo *K*α radiation, λ = 0.71073 Å
Hall symbol: P 2yb	Cell parameters from 982 reflections
*a* = 8.6313 (13) Å	θ = 2.8–21.4°
*b* = 4.9373 (8) Å	µ = 0.25 mm^−^^1^
*c* = 19.610 (3) Å	*T* = 295 K
β = 102.359 (3)°	Block, colourless
*V* = 816.3 (2) Å^3^	0.25 × 0.17 × 0.12 mm
*Z* = 2	

### Data collection

**Table:**

Bruker APEXII CCD area-detector diffractometer	2837 independent reflections
Radiation source: fine-focus sealed tube	2047 reflections with *I* > 2σ(*I*)
graphite	*R*_int_ = 0.024
φ and ω scans	θ_max_ = 25.0°, θ_min_ = 1.1°
Absorption correction: multi-scan (*SADABS*; Sheldrick, 2003)	*h* = −10→7
*T*_min_ = 0.940, *T*_max_ = 0.971	*k* = −5→5
4350 measured reflections	*l* = −15→23

### Refinement

**Table:**

Refinement on *F*^2^	Secondary atom site location: difference Fourier map
Least-squares matrix: full	Hydrogen site location: inferred from neighbouring sites
*R*[*F*^2^ > 2σ(*F*^2^)] = 0.043	H-atom parameters constrained
*wR*(*F*^2^) = 0.098	*w* = 1/[σ^2^(*F*_o_^2^) + (0.0334*P*)^2^ + 0.0867*P*] where *P* = (*F*_o_^2^ + 2*F*_c_^2^)/3
*S* = 1.05	(Δ/σ)_max_ < 0.001
2837 reflections	Δρ_max_ = 0.13 e Å^−^^3^
209 parameters	Δρ_min_ = −0.19 e Å^−^^3^
1 restraint	Absolute structure: Flack (1983), 1207 Friedel pairs
Primary atom site location: structure-invariant direct methods	Flack parameter: 0.13 (9)

### Special details

**Table:**

Geometry. All e.s.d.'s (except the e.s.d. in the dihedral angle between two l.s. planes) are estimated using the full covariance matrix. The cell e.s.d.'s are taken into account individually in the estimation of e.s.d.'s in distances, angles and torsion angles; correlations between e.s.d.'s in cell parameters are only used when they are defined by crystal symmetry. An approximate (isotropic) treatment of cell e.s.d.'s is used for estimating e.s.d.'s involving l.s. planes.
Refinement. Refinement of *F*^2^ against ALL reflections. The weighted *R*-factor *wR* and goodness of fit *S* are based on *F*^2^, conventional *R*-factors *R* are based on *F*, with *F* set to zero for negative *F*^2^. The threshold expression of *F*^2^ > σ(*F*^2^) is used only for calculating *R*-factors(gt) *etc*. and is not relevant to the choice of reflections for refinement. *R*-factors based on *F*^2^ are statistically about twice as large as those based on *F*, and *R*- factors based on ALL data will be even larger.

### Fractional atomic coordinates and isotropic or equivalent isotropic displacement parameters (Å^2^)

**Table:**

	*x*	*y*	*z*	*U*_iso_*/*U*_eq_	
Cl1	0.70397 (10)	0.5956 (2)	0.50973 (4)	0.0773 (3)	
O1	0.5785 (2)	0.0599 (5)	0.75880 (12)	0.0668 (6)	
H1	0.5050	0.1302	0.7727	0.100*	
O2	0.2166 (3)	0.0098 (4)	0.82097 (12)	0.0690 (7)	
O3	−0.3408 (3)	0.4662 (5)	0.95022 (12)	0.0740 (7)	
N1	0.2158 (3)	0.4370 (5)	0.78114 (13)	0.0541 (7)	
H1A	0.1733	0.5953	0.7763	0.065*	
N2	0.3433 (3)	0.3712 (5)	0.75179 (13)	0.0528 (7)	
C1	0.6042 (4)	0.1967 (6)	0.70286 (16)	0.0529 (8)	
C2	0.5080 (3)	0.4145 (6)	0.67236 (15)	0.0451 (7)	
C3	0.5434 (3)	0.5345 (7)	0.61244 (14)	0.0494 (8)	
H3	0.4814	0.6771	0.5909	0.059*	
C4	0.6678 (4)	0.4446 (7)	0.58540 (16)	0.0552 (9)	
C5	0.7620 (4)	0.2322 (8)	0.61561 (19)	0.0635 (9)	
H5	0.8454	0.1714	0.5965	0.076*	
C6	0.7307 (3)	0.1117 (8)	0.67442 (18)	0.0639 (9)	
H6	0.7951	−0.0289	0.6956	0.077*	
C7	0.3708 (3)	0.5104 (6)	0.69966 (15)	0.0452 (7)	
C8	0.1611 (4)	0.2390 (7)	0.81801 (16)	0.0520 (8)	
C9	0.0293 (3)	0.3162 (6)	0.85165 (14)	0.0445 (7)	
C10	−0.0777 (4)	0.5202 (7)	0.82750 (16)	0.0574 (9)	
H10	−0.0642	0.6230	0.7895	0.069*	
C11	−0.2041 (3)	0.5764 (7)	0.85793 (15)	0.0587 (8)	
H11	−0.2762	0.7119	0.8401	0.070*	
C12	−0.2215 (4)	0.4274 (7)	0.91544 (15)	0.0525 (8)	
C13	−0.1146 (4)	0.2243 (7)	0.94116 (16)	0.0577 (9)	
H13	−0.1264	0.1251	0.9800	0.069*	
C14	0.0089 (4)	0.1689 (6)	0.90945 (15)	0.0544 (8)	
H14	0.0799	0.0310	0.9268	0.065*	
C15	0.2677 (3)	0.7379 (7)	0.66588 (15)	0.0536 (8)	
H15A	0.3300	0.8609	0.6440	0.064*	
H15B	0.2283	0.8380	0.7012	0.064*	
C16	0.1279 (4)	0.6325 (11)	0.61111 (19)	0.0939 (13)	
H16A	0.1665	0.5444	0.5744	0.141*	
H16B	0.0608	0.7813	0.5921	0.141*	
H16C	0.0684	0.5056	0.6323	0.141*	
C17	−0.4560 (4)	0.6733 (9)	0.9258 (2)	0.0895 (13)	
H17A	−0.4043	0.8464	0.9289	0.134*	
H17B	−0.5342	0.6742	0.9540	0.134*	
H17C	−0.5065	0.6377	0.8780	0.134*	

### Atomic displacement parameters (Å^2^)

**Table:**

	*U*^11^	*U*^22^	*U*^33^	*U*^12^	*U*^13^	*U*^23^
Cl1	0.0774 (6)	0.0948 (7)	0.0677 (5)	−0.0054 (6)	0.0335 (4)	−0.0015 (6)
O1	0.0718 (15)	0.0543 (16)	0.0764 (14)	0.0118 (13)	0.0205 (12)	0.0138 (14)
O2	0.1008 (18)	0.0361 (15)	0.0827 (16)	0.0085 (12)	0.0477 (15)	0.0034 (12)
O3	0.0694 (15)	0.085 (2)	0.0769 (16)	0.0074 (14)	0.0363 (13)	0.0030 (14)
N1	0.0677 (17)	0.0358 (15)	0.0667 (16)	0.0042 (13)	0.0319 (14)	0.0035 (13)
N2	0.0639 (17)	0.0403 (16)	0.0606 (16)	0.0001 (13)	0.0278 (14)	0.0000 (14)
C1	0.0542 (19)	0.041 (2)	0.062 (2)	−0.0043 (16)	0.0091 (17)	−0.0025 (17)
C2	0.0453 (17)	0.0380 (17)	0.0522 (18)	−0.0049 (14)	0.0108 (14)	−0.0051 (16)
C3	0.0473 (17)	0.050 (2)	0.0519 (17)	−0.0014 (16)	0.0116 (14)	−0.0026 (16)
C4	0.0522 (18)	0.059 (2)	0.0562 (19)	−0.0078 (18)	0.0162 (16)	−0.0090 (18)
C5	0.0491 (19)	0.056 (2)	0.089 (3)	0.0005 (19)	0.0211 (19)	−0.013 (2)
C6	0.0515 (18)	0.052 (2)	0.087 (2)	0.0091 (18)	0.0129 (17)	−0.004 (2)
C7	0.0491 (17)	0.0347 (17)	0.0529 (18)	−0.0037 (14)	0.0134 (15)	−0.0061 (15)
C8	0.069 (2)	0.0380 (19)	0.053 (2)	−0.0046 (18)	0.0224 (17)	−0.0020 (16)
C9	0.0575 (19)	0.0341 (17)	0.0447 (18)	−0.0021 (15)	0.0169 (15)	−0.0039 (14)
C10	0.069 (2)	0.053 (2)	0.0545 (18)	0.0021 (18)	0.0208 (16)	0.0090 (17)
C11	0.0565 (18)	0.057 (2)	0.0637 (19)	0.007 (2)	0.0151 (16)	0.001 (2)
C12	0.0558 (19)	0.055 (2)	0.0492 (18)	−0.0047 (18)	0.0173 (16)	−0.0069 (18)
C13	0.072 (2)	0.053 (2)	0.0514 (19)	0.0018 (19)	0.0227 (17)	0.0089 (17)
C14	0.069 (2)	0.046 (2)	0.0505 (18)	0.0060 (16)	0.0188 (16)	0.0054 (16)
C15	0.057 (2)	0.0478 (19)	0.062 (2)	0.0037 (17)	0.0244 (17)	0.0041 (17)
C16	0.056 (2)	0.113 (4)	0.106 (3)	−0.006 (2)	0.004 (2)	0.010 (3)
C17	0.074 (3)	0.099 (4)	0.104 (3)	0.013 (3)	0.039 (2)	−0.002 (3)

### Geometric parameters (Å, °)

**Table:**

Cl1—C4	1.747 (3)	C8—C9	1.481 (4)
O1—C1	1.346 (3)	C9—C10	1.380 (4)
O1—H1	0.8200	C9—C14	1.389 (4)
O2—C8	1.225 (4)	C10—C11	1.379 (4)
O3—C12	1.364 (3)	C10—H10	0.9300
O3—C17	1.435 (4)	C11—C12	1.381 (4)
N1—C8	1.359 (4)	C11—H11	0.9300
N1—N2	1.385 (3)	C12—C13	1.383 (4)
N1—H1A	0.8600	C13—C14	1.372 (4)
N2—C7	1.295 (3)	C13—H13	0.9300
C1—C6	1.393 (4)	C14—H14	0.9300
C1—C2	1.411 (4)	C15—C16	1.525 (4)
C2—C3	1.407 (4)	C15—H15A	0.9700
C2—C7	1.478 (4)	C15—H15B	0.9700
C3—C4	1.369 (4)	C16—H16A	0.9600
C3—H3	0.9300	C16—H16B	0.9600
C4—C5	1.381 (5)	C16—H16C	0.9600
C5—C6	1.375 (5)	C17—H17A	0.9600
C5—H5	0.9300	C17—H17B	0.9600
C6—H6	0.9300	C17—H17C	0.9600
C7—C15	1.497 (4)		
			
C1—O1—H1	109.5	C11—C10—C9	122.1 (3)
C12—O3—C17	118.4 (3)	C11—C10—H10	118.9
C8—N1—N2	116.7 (2)	C9—C10—H10	118.9
C8—N1—H1A	121.6	C10—C11—C12	118.7 (3)
N2—N1—H1A	121.6	C10—C11—H11	120.6
C7—N2—N1	120.0 (3)	C12—C11—H11	120.6
O1—C1—C6	117.1 (3)	O3—C12—C11	124.0 (3)
O1—C1—C2	122.9 (3)	O3—C12—C13	115.8 (3)
C6—C1—C2	120.0 (3)	C11—C12—C13	120.2 (3)
C3—C2—C1	117.5 (3)	C14—C13—C12	120.1 (3)
C3—C2—C7	120.2 (3)	C14—C13—H13	119.9
C1—C2—C7	122.3 (3)	C12—C13—H13	119.9
C4—C3—C2	121.1 (3)	C13—C14—C9	120.8 (3)
C4—C3—H3	119.5	C13—C14—H14	119.6
C2—C3—H3	119.5	C9—C14—H14	119.6
C3—C4—C5	121.2 (3)	C7—C15—C16	111.2 (3)
C3—C4—Cl1	119.2 (3)	C7—C15—H15A	109.4
C5—C4—Cl1	119.5 (3)	C16—C15—H15A	109.4
C6—C5—C4	119.0 (3)	C7—C15—H15B	109.4
C6—C5—H5	120.5	C16—C15—H15B	109.4
C4—C5—H5	120.5	H15A—C15—H15B	108.0
C5—C6—C1	121.2 (3)	C15—C16—H16A	109.5
C5—C6—H6	119.4	C15—C16—H16B	109.5
C1—C6—H6	119.4	H16A—C16—H16B	109.5
N2—C7—C2	114.3 (3)	C15—C16—H16C	109.5
N2—C7—C15	123.8 (3)	H16A—C16—H16C	109.5
C2—C7—C15	121.8 (3)	H16B—C16—H16C	109.5
O2—C8—N1	120.8 (3)	O3—C17—H17A	109.5
O2—C8—C9	123.1 (3)	O3—C17—H17B	109.5
N1—C8—C9	116.0 (3)	H17A—C17—H17B	109.5
C10—C9—C14	118.0 (3)	O3—C17—H17C	109.5
C10—C9—C8	123.8 (3)	H17A—C17—H17C	109.5
C14—C9—C8	118.1 (3)	H17B—C17—H17C	109.5
			
C8—N1—N2—C7	158.5 (3)	N2—N1—C8—O2	−4.4 (5)
O1—C1—C2—C3	−177.6 (3)	N2—N1—C8—C9	177.4 (3)
C6—C1—C2—C3	1.3 (4)	O2—C8—C9—C10	−151.6 (3)
O1—C1—C2—C7	0.1 (4)	N1—C8—C9—C10	26.5 (4)
C6—C1—C2—C7	179.0 (3)	O2—C8—C9—C14	26.0 (5)
C1—C2—C3—C4	−0.7 (4)	N1—C8—C9—C14	−155.8 (3)
C7—C2—C3—C4	−178.5 (3)	C14—C9—C10—C11	−1.4 (4)
C2—C3—C4—C5	0.5 (5)	C8—C9—C10—C11	176.3 (3)
C2—C3—C4—Cl1	178.6 (2)	C9—C10—C11—C12	1.6 (5)
C3—C4—C5—C6	−0.8 (5)	C17—O3—C12—C11	0.3 (5)
Cl1—C4—C5—C6	−178.8 (3)	C17—O3—C12—C13	−179.7 (3)
C4—C5—C6—C1	1.3 (5)	C10—C11—C12—O3	179.4 (3)
O1—C1—C6—C5	177.4 (3)	C10—C11—C12—C13	−0.7 (5)
C2—C1—C6—C5	−1.6 (5)	O3—C12—C13—C14	179.6 (3)
N1—N2—C7—C2	−178.6 (2)	C11—C12—C13—C14	−0.3 (5)
N1—N2—C7—C15	−2.6 (4)	C12—C13—C14—C9	0.5 (5)
C3—C2—C7—N2	175.1 (3)	C10—C9—C14—C13	0.3 (4)
C1—C2—C7—N2	−2.6 (4)	C8—C9—C14—C13	−177.5 (3)
C3—C2—C7—C15	−1.0 (4)	N2—C7—C15—C16	−84.3 (4)
C1—C2—C7—C15	−178.7 (3)	C2—C7—C15—C16	91.4 (3)

### Hydrogen-bond geometry (Å, °)

**Table:**

*D*—H···*A*	*D*—H	H···*A*	*D*···*A*	*D*—H···*A*
O1—H1···N2	0.82	1.81	2.526 (3)	145
N1—H1A···O2^i^	0.86	2.23	2.934 (3)	140

Symmetry codes: (i) *x*, *y*+1, *z*.
